# Does the “Obesity Paradox” Have an Expiration Date? A Retrospective Cohort Study

**DOI:** 10.3390/jcm12216765

**Published:** 2023-10-26

**Authors:** Matan Elkan, Natalia Kofman, Sa’ar Minha, Nadav Rappoport, Ronit Zaidenstein, Ronit Koren

**Affiliations:** 1 Department of Internal Medicine A, Shamir Medical Center (Assaf Harofeh), Zerifin 7030000, Israelronitkoren@gmail.com (R.K.); 2 Department of Cardiology, Shamir Medical Center (Assaf Harofeh), Zerifin 7030000, Israel; 3 Faculty of Medicine, Tel Aviv University, Tel Aviv 6997801, Israel; 4 Department of Software and Information Systems Engineering, Ben-Gurion University of the Negev, Beer-Sheva 8410501, Israel; 5 Division of Government Medical Centers, Israeli Ministry of Health, Jerusalem 9101002, Israel

**Keywords:** body mass index, obesity paradox, infectious disease, hospital medicine, mortality

## Abstract

(1) Background: The “obesity paradox” refers to a protective effect of higher body mass index (BMI) on mortality in acute infectious disease patients. However, the long-term impact of this paradox remains uncertain. (2) Methods: A retrospective study of patients diagnosed with community-acquired acute infectious diseases at Shamir Medical Center, Israel (2010–2020) was conducted. Patients were grouped by BMI: underweight, normal weight, overweight, and obesity classes I–III. Short- and long-term mortality rates were compared across these groups. (3) Results: Of the 25,226 patients, diverse demographics and comorbidities were observed across BMI categories. Short-term (90-day) and long-term (one-year) mortality rates were notably higher in underweight and normal-weight groups compared to others. Specifically, 90-day mortality was 22% and 13.2% for underweight and normal weight respectively, versus 7–9% for others (*p* < 0.001). Multivariate time series analysis revealed underweight individuals had a significantly higher 5-year mortality risk (HR 1.41 (95% CI 1.27–1.58, *p* < 0.001)), while overweight and obese categories had a reduced risk (overweight—HR 0.76 (95% CI 0.72–0.80, *p* < 0.001), obesity class I—HR 0.71 (95% CI 0.66–0.76, *p* < 0.001), obesity class II—HR 0.77 (95% CI 0.70–0.85, *p* < 0.001), and obesity class III—HR 0.79 (95% CI 0.67–0.92, *p* = 0.003)). (4) Conclusions: In this comprehensive study, obesity was independently associated with decreased short- and long-term mortality. These unexpected results prompt further exploration of this counterintuitive phenomenon.

## 1. Introduction

Obesity is a chronic disease that affects millions globally. A major complication of obesity is type 2 diabetes mellitus (T2DM), which elevates the risk of cancer, cardiovascular disease (CVD), and kidney disease. Obesity is also associated with many other diseases, including heart failure, venous thromboembolism, hepatobiliary and liver disease, and chronic lung disease including obstructive sleep apnea and asthma [1,2]. The rising prevalence of obesity ranks it fourth among mortality risk factors, largely due to the influence of high body mass index (BMI) on CVD [3,4].

Being underweight can also be associated with chronic diseases, especially when it arises from unintentional weight loss. This association is further emphasized in the presence of conditions such as sarcopenia or cachexia. Both conditions are characterized by a loss of muscle mass and decreased functional performance, but cachexia additionally involves potential fat mass loss and disturbances in the homeostatic control of energy and protein balance [5,6]. Cachexia is frequently linked with malignancy [7] but can also be associated with other chronic diseases, including heart failure [8], liver cirrhosis [9], and chronic obstructive pulmonary disease (COPD) [10], among others.

The term “obesity paradox” refers to the observed favorable outcomes in overweight and obese patients across a variety of medical conditions. These conditions include CVD such as acute myocardial infarction [11] and heart failure [12], as well as cancer [13] and hip fractures [14]. Multiple explanations have been proposed for this advantageous aspect of obesity. Increased body weight may enable greater muscle and adipose reserve, improved endotoxin scavenging effects, a decrease in prothrombotic factors, and the production of favorable adipokines such as adiponectin, apelin, and omentin, among others [15]. However, the true nature of the obesity paradox remains debated. Some studies have suggested a survival bias, particularly where the paradox was not sustained in younger individuals [16]. Concerns have also arisen regarding potential confounding factors that could mistakenly attribute the observed effects to alternative risk factors [17]. Additionally, the potential presence of collider stratification bias and unidentified illnesses in hospitalized individuals of normal weight have raised further concerns [18].

The “obesity paradox” has also been observed in various infectious diseases, including pneumonia [19], soft tissue infections [20], and sepsis [21]. In these conditions, being overweight and obese has been linked with short-term survival benefits. A comprehensive Danish study of over 35,000 patients hospitalized for acute infectious diseases reinforced this observation. Within this cohort, overweight or obese patients displayed significantly reduced adjusted 90-day mortality rates (aHR 0.64 (95% CI, 0.58 to 0.69)) and (aHR 0.55 (95% CI, 0.49 to 0.62)) in comparison to their normal-weight counterparts. Conversely, underweight individuals exhibited a heightened mortality risk (aHR 1.75; (95% CI, 1.58 to 1.94)) [22].

Nevertheless, there is limited data on the long-term effects of the “obesity paradox”. While the continuity of the paradox was observed at three years post myocardial infarction [23] and one year after COPD exacerbation [24], it was not evident 10 years following coronary artery bypass grafting surgery [25]. In this study, we aim to investigate the short- and long-term effects of BMI on outcomes of inpatients admitted with community-acquired infectious diseases at a large medical center.

## 2. Materials and Methods

We retrospectively analyzed clinical data from patients admitted to Shamir Medical Center between the years 2010 and 2020. The data were accessed through the Israeli Ministry of Health Kineret Platform. The Kineret platform is a cloud-based service that facilitates the analysis and study of patient-level electronic health records (EHR) data. The data were anonymized and securely uploaded to a database in the Observational Medical Outcomes Partnership (OMOP) Common Data Model (CDM) [26]. We obtained the cohort design and description using ATLAS [27], an open-source web-based tool developed by the Observational Health Data Sciences and Informatics (OHDSI) community [28]. OHDSI is an international collaborative whose goal is to create and apply open-source data analytics solutions to help represent healthcare data from diverse sources in a consistent and standardized way, enabling multi-site studies without sharing raw data. Research involving human subjects complied with all relevant national regulations, institutional policies, was in accordance with the tenets of the Helsinki Declaration (as revised in 2013), and was approved by the authors. The study received approval from the local institutional ethics committee prior to its initiation.

### 2.1. Study Population

The study included consecutive adult patients (≥18 years) who were admitted to Shamir Medical Center from 1 January 2010 to 31 December 2020 due to a diagnosis of community-acquired infectious disease. We conducted a follow-up on all-cause mortality until 31 May 2022. Exclusion criteria included patients without a BMI measurement during the index visit and pregnant patients.

### 2.2. Data Collection and Definition

We defined all observations and diagnoses data using Systematized Nomenclature of Medicine Clinical Terms (SNOMED CT) codes, which serve as the core terminology within OMOP CDM. A list of infectious diseases and comorbidities included in this study and their definitions based on SNOMED CT codes can be found in Supplementary Tables S1 and S2. BMI was derived from EHR data at the index visit admission. Measurements were conducted by an experienced nurse or self-reported height and weight was utilized. Previous studies have shown that BMI based on self-reported data approximates measured BMI by about 4% and is thus considered reliable [29]. Laboratory indices, including serum hemoglobin, albumin, creatinine, and C-reactive protein (CRP), were collected and included the first available result after admission. All-cause mortality data were obtained from the national registry of Israel’s Ministry of Interior Affairs; specific-cause mortality was not available.

### 2.3. Statistical Analysis

The primary outcome was all-cause mortality within 90 days, one year, and five years. We tested the outcomes as a binary variable and in a time-dependent analysis. BMI was analyzed as an ordinal variable, where the normal range of BMI (18.5–24.9) was defined as the reference.

Univariate analysis: We employed the chi-squared test for binary variables and ANOVA for continuous variables to examine differences in individual features across the six BMI categories. The Kruskal–Wallis rank-sum test was employed to assess differences in outcomes based on the BMI category. After the Kruskal–Wallis test, a post hoc pairwise test for multiple comparisons was conducted using the dunn.test v1.3.5 R package. Dunn’s test with Bonferroni correction was applied to address the issue of multiple testing.

Survival analysis was performed using the Cox proportional hazards model. The adjusted multivariable analysis included the following predefined covariates. Demographic factors: age, gender, BMI categories. Laboratory results: albumin, creatinine, CRP, hemoglobin. Chronic comorbidities: T2DM, chronic lung disease, ischemic heart disease, past myocardial infarction, heart failure, malignancy, chronic kidney disease, metabolic syndrome, hypertension, hyperlipidemia. Acute infectious conditions: endocarditis, peritonitis, diverticulitis, appendicitis, cholangitis and cholecystitis, pneumonia, viral upper respiratory tract infection, ear, nose, and throat infections, central nervous system infections, necrotizing fasciitis, infective arthritis, osteomyelitis, cellulitis, urinary tract infection. All Analyses were done using R v4.1.2.3.

## 3. Results

Among 45,115 patients admitted with a community-acquired infectious disease diagnosis, 25,226 (55.9%) had a documented BMI measurement. Patient demographics, comorbidities, and laboratory results are presented in Table 1. A total of 49.6% were female and the mean age was 61.6 ± 21.2. The underweight BMI category included 815 (3.2%) patients, while the normal BMI category had 9307 (36.9%). The overweight category comprised 8702 patients (34.5%), and there were 4179 (16.6%), 1612 (6.4%), and 611 (2.4%) patients in obesity classes I, II, and III, respectively.

Patients in the various BMI categories exhibited significant diversity across all parameters. The prevalence of the female sex was higher in both the underweight and all obesity categories: 59.6% in the underweight category and 51.7%, 60.9%, and 61.7% in obesity classes II, II, and III respectively. Generally, the prevalence of comorbidities increased with higher BMI categories, with one notable exception: the prevalence of malignancy was highest in the underweight category and lowest in obesity class III. The prevalence of different infectious disease diagnoses at index hospitalization is presented in Table 1.

The incidence of mortality at the 90-day, one, and five-year follow-ups is presented in Table 2. Ninety-day mortality was 22.0% in the underweight category, 13.2% in normal weight, 8.9% in overweight, and 7.2%, 9.0%, and 7.0% in obesity class I, II, and III, respectively (*p* < 0.001). One-year mortality was 31.8% in the underweight category, 20.6% in normal weight, 15.6% in overweight, and 13.0%, 15.0%, and 15.1% in obesity class I, II, and III, respectively (*p* < 0.001). Five-year mortality was 44.4% in the underweight category, 32.8% in normal weight, 29.4% in overweight, and 26.4%, 30.2%, and 27.3% in obesity class I, II, and III respectively (*p* < 0.001). Similar mortality rates were observed in the overall population of patients following hospitalization for community-acquired infectious disease, including those with no BMI measurement.

Post hoc analysis indicated that for 90-day and one-year mortality, only the overweight and normal weight categories showed significant differences from each other and all other categories. For five-year mortality, underweight was the only category significantly different from all other categories.

### Time-Dependent Analysis

**Figure 1:**
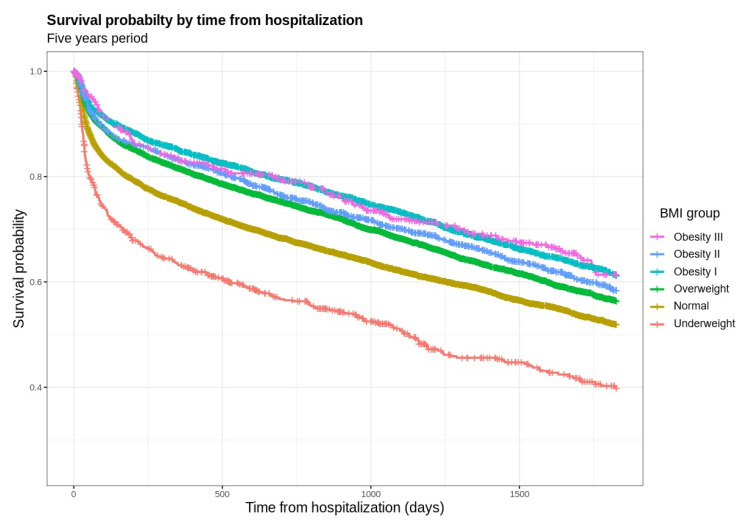
Survival probability by BMI group up to 5–years after hospitalization for an infectious disease. Underweight BMI 15–18.49, Normal 18.5–24.9, Overweight 25–29.9, Obesity I 30–34.9, Obesity II 35–39.9, Obesity III 40–45.

**Figure 2:**
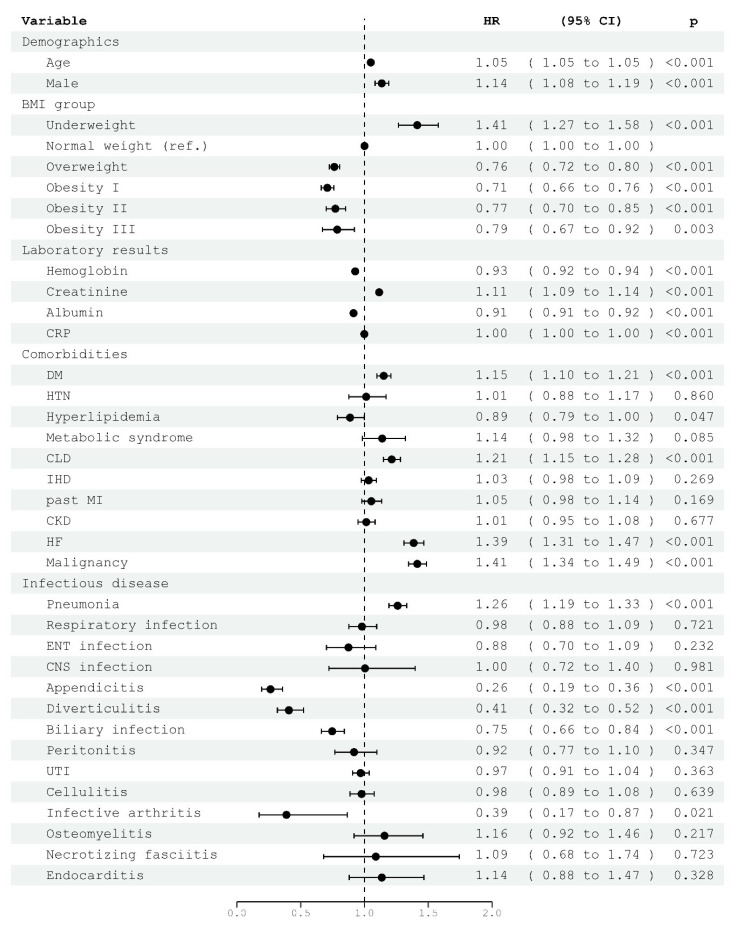
Forest plot of Cox proportional hazard multivariable modeling on survival five years after hospitalization for infectious disease, including BMI categories and covariates. DM—diabetes mellitus, CLD—chronic lung disease, IHD—ischemic heart disease, MI—myocardial infarction, HF—heart failure, CKD—chronic kidney disease, HTN—hypertension, MetS—metabolic syndrome, CRP—C-reactive protein, SD—standard deviation. URTI—upper respiratory tract infection, ENT—ear, nose, and throat, UTI—urinary tract infection. Biliary infection includes cholecystitis and cholangitis.

To examine the impact of BMI category on the probability of survival up to five years after index hospitalization, we employed a Cox proportional hazard model. Comparisons of survival curves are presented in Figure 1. Results adjusted for covariates including demographics, comorbidities, infectious disease, and lab results are presented in Figure 2. Significant differences in survival were observed among patients in all BMI categories when compared to those with normal weight. At five years, patients in the underweight category exhibited a decreased probability of survival, hazard ratio (HR) 1.41 (95% CI 1.27–1.58, *p* < 0.001). Conversely, patients in the overweight and obesity categories showed an increased probability of survival; overweight patients had an HR of 0.76 (95% CI 0.72–0.80, *p* < 0.001), obesity class I had an HR of 0.71 (95% CI 0.66–0.76, *p* < 0.001), obesity class II had an HR of 0.77 (95% CI 0.70–0.85, *p* < 0.001), and obesity class III had an HR of 0.79 (95% CI 0.67–0.92, *p* = 0.003). To avoid potential bias, due to the high proportion of patients with malignancy in the underweight category, we completed a second series of analysis, excluding patients with no substantial difference in the results (Supplementary Tables S3 and S4).

## 4. Discussion

This observational longitudinal study examined the relationship between BMI and mortality in 25,226 hospitalized patients with an acute infectious disease. Our findings revealed that being underweight or normal weight were independent risk factors for mortality as compared to being overweight or obese over a five-year span. The detrimental effect of being underweight on survival was evident in both univariate and multivariate analysis, aligning with prior studies on the “obesity paradox”. Increased mortality risks were also associated with specific infectious causes (like pneumonia, necrotizing fasciitis, and endocarditis), as well as certain comorbidities (notably heart failure and malignancy) and laboratory test results (such as albumin, creatinine, and hemoglobin levels).

The overall mortality rate in our study population was substantial; 90-day mortality was 10.6% and one-year and five-year mortality were 17.5% and 31.2%, respectively. Similar short-term results have been reported previously. In a large Danish cohort of 35,406 patients hospitalized for acute infection, 90-day mortality was 9.8% for all patients, 23.6% in the underweight category, 12.2% in the normal weight category, and 7.3% and 5.1% in the overweight and obesity categories, respectively [22]. The results of this study may be influenced by the high percentage of patients admitted with community-acquired pneumonia (CAP), which accounted for 26% of the patients. CAP has previously been shown to be associated with increased long-term mortality; for example, in one population-based study that evaluated 7449 patients hospitalized with CAP, one-year mortality was 31% [30].

The greatest impact on mortality after multivariable analysis was attributed to being underweight, along with having malignancy or chronic heart failure. Being underweight might reflect unintentional weight loss or severe underlying comorbidities, which are sometimes unknown, and has been previously linked to increased mortality [15,31,32]. Heart failure has also previously been shown to be an important prognostic factor in patients with acute infection [33]. Previous studies assessing the “obesity paradox” have also found an increased risk of mortality in patients with malignancy and CVD, especially in patients admitted with pneumonia [34,35,36]. Some studies have also shown an increased risk for patients with COPD and chronic kidney disease [37], while other studies have not shown this increased risk [34,35].

Considering that obesity is a risk factor for infections as well as a range of chronic illnesses, its association with decreased mortality in the acute setting is noteworthy. Several previous epidemiological studies have shown favorable outcomes for individuals with higher BMI levels. Specifically, outcomes for obese patients with various infectious diseases, such as pneumonia, tuberculosis, peritonitis, and sepsis, were significantly better compared to those with normal BMI levels [38]. However, a recent exception to this trend can be observed in patients with COVID-19. In several studies, obesity was identified as a risk factor for severe disease progression and even increased mortality, thereby challenging the notion of an “obesity paradox” for this particular illness [39,40].

Several explanations have been proposed in the past to elucidate this phenomenon, with two main approaches: a true protective effect due to metabolic reserve or immunologic modulations, and a false paradox caused by epidemiological bias. Obesity may enhance the ability to cope with acute illness by providing greater adipose reserve, improved endotoxin scavenging effects, decrease in prothrombotic factors, and production of favorable adipokines [15]. Interestingly, animal studies have demonstrated a beneficial effect on survival from infectious diseases through an increased-fat diet that promotes obesity [41,42]. These preliminary studies suggest a potential immune-protective role of leptin [43].

Other factors to consider are the potential biases that obese patients might encounter both prior to and during hospitalization, including a reduced index of suspicion and a propensity for over diagnosing infectious diseases [44]. While these biases cannot be entirely ruled out in our study, it is notable that CRP levels were lowest in individuals with a normal BMI. There may also be a tendency to initiate antibiotic treatments more readily in obese patients due to their associated comorbidities. Yet, even after adjusting for these potential confounders, our findings remained significant. In addition, a theory has been posited that infectious diseases linked to obesity, while potentially more common, could be less severe than in patients with other chronic diseases, creating a perception of a protective advantage [45].

In this study, 15,104 patients were overweight and obese, accounting for 59% of the study population. According to the National Program for Quality Indicators in Community Healthcare in Israel for the period 2017–2019 [31], this study found a lower prevalence of obesity among the following age groups of inpatients: 64–69 years (34.1% vs. 34.5%), 70–74 years (30.8% vs. 33.8%) 75–80 years (28% vs. 31.5%), and 80–84 years (26.6% vs. 30.5%). Among younger patients, a higher proportion of outpatients had normal weight compared to inpatients. Considering that obesity is a risk factor for both nosocomial and community-acquired infections [32,33], it would be reasonable to anticipate higher rates of obese patients in this study population, compared to healthy individuals. Their under-representation in this study cohort may partially explain the observed paradox. Patients with a normal BMI requiring hospitalization may have more severe acute illness or an unknown underlining disease causing unintentional weight loss prior to their index hospitalization. Additionally, the possibility exists that overweight and obese inpatients might be metabolically different from obese outpatients, especially among the elderly.

The study findings suggest that the protective effect of obesity persists in the long term, lasting up to five years after index hospitalization. To the best of our knowledge, this is the first study to provide evidence of an “obesity paradox” five years after an acute infectious disease [46]. These findings contribute to previous studies that primarily had short-term follow-up available [19,47,48], as well as to a study of patients with severe sepsis demonstrating survival benefits in obese patients at one year [49].

### Study Limitations

This retrospective large-scale study has inherent limitations due to its observational nature. BMI measurements were available for only 55.9% of patients, a limitation that is shared by previous retrospective studies from different cohorts [14]. In addition, our study relied on self-reported BMI; while this method has been shown to be a good approximation to measured BMI [29], it may still be influenced by biases, for example different psychological dispositions that may influence reporting [50]. There are important risk factors that we were unable to collect from our data and might be important for interpretating patients’ metabolic status. For instance, we lack information about patients’ nutritional habits, whether individuals classified as “normal weight” are genuinely metabolically healthy or if they recently experienced weight loss or have an undiagnosed illness. Furthermore, BMI may not be the optimal parameter to assess adiposity. Other measures such as waist-to-hip ratio or waist circumference might better predict body composition. It is also worth considering that normal weight patients, especially the elderly, may be sarcopenic.

Another limitation is the lack of data regarding parameters of the acute infectious syndromes, including the exact pathogen, timing, or treatment regimen and other acute interventions that could impact the results.

## 5. Conclusions

Our study emphasizes the significant impact of the “obesity paradox” on the survival of patients up to five years following hospitalization due to acute infections. Future prospective research should further explore this captivating phenomenon, particularly highlighting factors like body composition (taking into account both fat distribution and lean mass quantity), cardiovascular fitness, the duration of obesity, fat-associated inflammatory markers, and the differential outcomes of ambulatory as compared to hospitalized patients.

## Figures and Tables

**Table 1:** Patient demographics, comorbidities, basic laboratory results, and acute infectious diagnosis at index hospitalization.

BMI Categories	Underweight 15–18.49	Normal 18.5–24.9	Overweight 25–29.9	Obesity I 30–34.9	Obesity II 35–39.9	Obesity III 40–45	*p*
N = 25,226	815	9307	8702	4179	1612	611	
Age, mean (SD)	57.16 (25.0)	58.24 (24.0)	63.41 (19.6)	63.68 (18.2)	63.15 (17.7)	61.64 (16.6)	<0.001
Female, n (%)	486 (59.6)	4633 (49.8)	3873 (44.5)	2160 (51.7)	982 (60.9)	377 (61.7)	<0.001
Comorbidities							
DM, n (%)	107 (13.1)	1724 (18.5)	2400 (27.6)	1397 (33.4)	666 (41.3)	276 (45.2)	<0.001
CLD, n (%)	152 (18.7)	1164 (12.5)	1210 (13.9)	695 (16.6)	363 (22.5)	155 (25.4)	<0.001
IHD, n (%)	94 (11.5)	1363 (14.6)	1669 (19.2)	816 (19.5)	318 (19.7)	130 (21.3)	<0.001
HF, n (%)	71 (8.7)	926 (9.9)	1146 (13.2)	591 (14.1)	315 (19.5)	142 (23.2)	<0.001
Malignancy, n (%)	163 (20.0)	1450 (15.6)	1379 (15.8)	621 (14.9)	245 (15.2)	69 (11.3)	<0.001
CKD, n (%)	59 (7.2)	762 (8.2)	830 (9.5)	407 (9.7)	193 (12.0)	72 (11.8)	<0.001
HTN, n (%)	29 (3.6)	512 (5.5)	712 (8.2)	419 (10.0)	159 (9.9)	64 (10.5)	<0.001
Hyperlipidemia, n (%)	26 (3.2)	359 (3.9)	545 (6.3)	295 (7.1)	122 (7.6)	45 (7.4)	<0.001
MetS, n (%)	50 (6.1)	685 (7.4)	910 (10.5)	492 (11.8)	201 (12.5)	68 (11.1)	<0.001
Laboratory results							
Albumin, mean (SD)	32.4 (6.3)	34.1 (5.6)	34.6 (5.2)	35.1 (4.8)	34.5 (4.8)	34.9 (4.6)	<0.001
Creatinine, mean (SD)	1.0 (0.91)	1.11 (1.00)	1.16 (0.95)	1.19 (0.93)	1.24 (1.09)	1.21 (1.06)	<0.001
CRP, mean (SD)	104 (99)	95 (93)	98 (94)	100 (97)	104 (103)	107 (103)	<0.001
Hemoglobin, mean (SD)	12.0 (2.0)	12.4 (2.1)	12.6 (2.1)	12.6 (2.0)	12.3 (2.1)	12.4 (2.0)	<0.001
Acute infectious diagnosis							
Pneumonia, n (%)	267 (32.8)	2416 (26.0)	2297 (26.4)	1051 (25.1)	370 (23.0)	132 (21.6)	<0.001
Viral URTI, n (%)	32 (3.9)	475 (5.1)	578 (6.6)	309 (7.4)	108 (6.7)	48 (7.9)	<0.001
Peritonitis, n (%)	21 (2.6)	139 (1.5)	126 (1.4)	41 (1.0)	19 (1.2)	9 (1.5)	0.012
Diverticulitis, n (%)	9 (1.1)	202 (2.2)	324 (3.7)	156 (3.7)	42 (2.6)	17 (2.8)	<0.001
Appendicitis, n (%)	61 (7.5)	1150 (12.4)	727 (8.4)	280 (6.7)	94 (5.8)	16 (2.6)	<0.001
Biliary infection *, n (%)	11 (1.3)	400 (4.3)	528 (6.1)	235 (5.6)	93 (5.8)	30 (4.9)	<0.001
Septic arthritis, n (%)	0 (0.0)	4 (0.0)	12 (0.1)	5 (0.1)	0 (0.0)	1 (0.2)	0.174
Osteomyelitis, n (%)	10 (1.2)	55 (0.6)	57 (0.7)	10 (0.2)	11 (0.7)	2 (0.3)	0.006
Cellulitis, n (%)	30 (3.7)	529 (5.7)	639 (7.3)	387 (9.3)	212 (13.2)	87 (14.2)	<0.001
Necrotizing fasciitis, n (%)	2 (0.2)	6 (0.1)	10 (0.1)	6 (0.1)	5 (0.3)	1 (0.2)	0.121
UTI, n (%)	151 (18.5)	1637 (17.6)	1451 (16.7)	718 (17.2)	241 (15.0)	72 (11.8)	0.001
CNS infection, n (%)	14 (1.7)	169 (1.8)	103 (1.2)	35 (0.8)	14 (0.9)	2 (0.3)	<0.001
ENT infection, n (%)	45 (5.5)	602 (6.5)	391 (4.5)	194 (4.6)	70 (4.3)	32 (5.2)	<0.001
Endocarditis, n (%)	5 (0.6)	47 (0.5)	51 (0.6)	18 (0.4)	7 (0.4)	3 (0.5)	0.876

CKD—chronic kidney disease, CLD—chronic lung disease, CNS—central nervous system, CRP—C-reactive protein, DM—diabetes mellitus, ENT—ear, nose, and throat, HF—heart failure, HTN—hypertension, IHD—ischemic heart disease, MetS—metabolic syndrome, SD—standard deviation, URTI—upper respiratory tract infection, UTI—urinary tract infection. * Biliary infection includes cholecystitis or cholangitis. Chi-squared test was applied to examine the differences in binary variables and ANOVA was applied to examine continuous variables.

**Table 2:** Mortality at 90-day, one-year, and five-year follow-up.

BMI Categories	Underweight 15–18.49	Normal 18.5–24.9	Overweight 25–29.9	Obesity I 30–34.9	Obesity II 35–39.9	Obesity III 40–45	*p*
N	815	9307	8702	4179	1612	611	
90-day, n (%)	179 (22.0)	1227 (13.2)	774 (8.9)	300 (7.2)	145 (9.0)	43 (7.0)	<0.001 ^a^
One-year, n (%)	259 (31.8)	1916 (20.6)	1356 (15.6)	544 (13.0)	242 (15.0)	92 (15.1)	<0.001 ^a^
Five-year, n (%)	362 (44.4)	3052 (32.8)	2556 (29.4)	1103 (26.4)	487 (30.2)	167 (27.3)	<0.001 ^b^

Kruskal–Wallis rank-sum test was employed to assess differences in outcomes based on the BMI category. In post hoc analysis, ^a^—underweight and normal weight were significantly different from each other and all other categories, ^b^—only underweight was significantly different from all other categories.

## Data Availability

Restrictions apply to the availability of these data. Data were obtained through the Kineret project of the Division of Government Medical Centers, in the Israeli Ministry of Health. For more information and data availability, please contact the corresponding author.

## Supplementary Materials

The following supporting information can be downloaded at: https://www.mdpi.com/article/10.3390/jcm12216765/s1, Table S1: List of acute infectious diseases included in analysis by SNOMED code; Table S2: List of chronic medical conditions included in analysis by SNOMED code. Table S3: Descriptive statistics and outcomes. Analysis without underweight patients. Table S4: Survival probability by BMI group up to 5-years after hospitalization with an infectious disease. Analysis without underweight patients.
