# Supplementary material for: Does the “Obesity Paradox” Have an Expiration Date? A Retrospective Cohort Study
Source: J Clin Med. 2023 Oct 26;12(21):6765. doi: 10.3390/jcm12216765 (PMC10647762; doi:10.3390/jcm12216765)
Supplement: Supplementary file 1 [file jcm-12-06765-s001.zip › jcm-2660029-supplementary.pdf]

Supplementary Materials

Table of contents:

Title: DOES THE “OBESITY PARADOX” HAVE AN EXPIRATION DATE? A RETROSPECTIVE COHORT STUDY.

Authors: Matan Elkan, Natalia Kofman, Sa'ar Minha, Nadav Rappoport, Ronit Zaidenstein, Ronit Koren.

Abbreviations and definitions…………………………………………………………2

Table S1…………………………………………………………………………………3

Table S2………………………………………………………………………..………..7

Table S3…………………………………………………………..……………………..20

Table S4…………………………………………………………..……………….....21

Abbreviations and definitions

Biliary infection- including cholecystitis or cholangitis

CKD- chronic kidney disease

CLD- chronic lung disease

CNS- Central nervous system

CRP- C-reactive protein

DM- diabetes mellitus

ENT- Ear, nose and throat

HF- heart failure

HTN- hypertension

IHD- ischemic heart disease

MetS- Metabolic syndrome

SD- standard deviation

URTI- Upper respiratory tract infection

UTI- Urinary tract infection

Table S1: List of acute infectious diseases included in analysis by SNOMED codes.

Name	SNOMED Code
Acute and subacute endocarditis	194921005
Acute and subacute infective endocarditis associated with another disorder	194926000
Acute appendicitis	85189001
Acute appendicitis with generalized peritonitis	28845006
Acute appendicitis with peritoneal abscess	51036000
Acute appendicitis without peritonitis	72048003
Acute bronchitis	10509002
Acute cholecystitis	65275009
Acute endocarditis	91357005
Acute epiglottitis with obstruction	222008
Acute ethmoidal sinusitis	67832005
Acute frontal sinusitis	91038008
Acute generalized peritonitis	52890006
Acute laryngopharyngitis	55355000
Acute maxillary sinusitis	68272006
Acute mucoid otitis media	52353000
Acute nonparalytic poliomyelitis	14535005
Acute non-suppurative otitis media - serous	194240006
Acute osteomyelitis	409780002
Acute osteomyelitis of ankle and/or foot	268017000
Acute osteomyelitis of hand	10188004
Acute osteomyelitis of humerus	203164005
Acute osteomyelitis of lower leg	203153007
Acute osteomyelitis of multiple sites	59867002
Acute osteomyelitis of pelvic region and/or thigh	268016009
Acute osteomyelitis of shoulder region	7587000
Acute otitis externa	30250000
Acute paralytic poliomyelitis	240460008
Acute paralytic poliomyelitis due to Human poliovirus 1	446958005
Acute paralytic poliomyelitis, bulbar	64764001
Acute peritonitis	67602004
Acute pharyngitis	363746003
Acute poliomyelitis	398102009
Acute pyelonephritis	36689008
Acute pyelonephritis with medullary necrosis	197769007
Acute pyelonephritis without medullary necrosis	197768004
Acute pyonephrosis with renal medullary necrosis	7448003
Acute sanguinous otitis media	77478005
Acute sinusitis	15805002

Acute sphenoidal sinusitis	77919000
Acute suppurative otitis media due to another disease	194282005
Acute suppurative otitis media with spontaneous rupture of ear drum	86279000
Acute tonsillitis	17741008
Acute upper respiratory infection	54398005
Amebic brain abscess	27908001
Appendicitis	74400008
Arthritis of knee due to viral infection	429669000
Bacterial enteritis	75375008
Bacterial meningitis	95883001
Bacterial meningitis due to Gram-negative bacteria	425887005
Bacterial pneumonia	53084003
Bile duct calculus with acute cholecystitis	197389006
Bile duct calculus with acute cholecystitis and obstruction	197391003
Bile peritonitis	36746002
Bronchitis caused by COVID-19	OMOP4873909
Bronchopneumonia	396285007
Calculus of bile duct with acute cholecystitis without obstruction	60127009
Calculus of bile duct with cholecystitis	36483003
Calculus of gallbladder and bile duct with acute cholecystitis	435131000124107
Calculus of gallbladder with acute cholecystitis	59771005
Calculus of gallbladder with cholecystitis	25924004
Candidiasis of lung	3487004
Cellulitis	128045006
Cellulitis and abscess of buttock	200676005
Cellulitis and abscess of face	200645004
Cellulitis and abscess of finger	200630006
Cellulitis and abscess of finger and toe	200628009
Cellulitis and abscess of forearm	200670004
Cellulitis and abscess of lower leg	200681001
Cellulitis and abscess of neck	267779003
Cellulitis and abscess of toe	200638004
Cellulitis and abscess of trunk	200655000
Cellulitis of leg, excluding foot	287001000
Chlamydial peritonitis	197172005
Cholangitis	82403002
Cholecystitis	76581006
Cholelithiasis AND cholecystitis with obstruction	50450007
Cholelithiasis AND cholecystitis without obstruction	29484002
Colitis, enteritis and gastroenteritis presumed infectious	266081001
COVID-19	840539006
Coxsackie meningitis	111878007
Diverticulitis of colon	111359004
Diverticulitis of small intestine	56165008
Echovirus meningitis	192667007
Elbow pyogenic arthritis	239779001
Empyema	312682007

Encephalitis caused by tick-borne encephalitis virus	712986001
Endocarditis	56819008
Endocarditis associated with another disorder	195012000
Erysipelas	44653001
Gallbladder calculus with acute cholecystitis and no obstruction	197377009
Gallbladder calculus with acute cholecystitis and obstruction	197378004
Gonococcal infection of joint	44743006
Gonococcal peritonitis	186939000
Group B streptococcal pneumonia	195886008
Haemophilus influenzae pneumonia	70036007
Haemophilus meningitis	192643004
Herpes simplex meningitis	23291008
Herpes zoster with meningitis	10491005
Herpetic meningoencephalitis	9678009
Infection of bone	111253001
Infection of bone associated with another disease	111252006
Infection of bone of ankle and/or foot	442449006
Infection of bone of hand	445012003
Infection of bone of pelvic region and/or femur	445013008
Infection of bone of shoulder girdle	444785006
Infection of bone of tibia and/or fibula	445215005
Infectious disorder of kidney	129128006
Infectious peritonitis	129129003
Infective arthritis	396234004
Infective arthritis of ankle and/or foot	444999002
Infective arthritis of elbow	445495007
Infective arthritis of joint of hand	428385007
Infective arthritis of pelvis	444998005
Infective arthritis of shoulder region	428437005
Infective meningitis	312216007
Infective otitis externa	86981007
Infective pneumonia	312342009
Influenza	6142004
Influenza due to Influenza A virus	442438000
Influenza with non-respiratory manifestation	61700007
Knee pyogenic arthritis	239777004
Legionella pneumonia	195889001
Meningococcal meningitis	192644005
Mumps meningitis	44201003
Mycoplasma pneumonia	46970008
Necrotizing fasciitis	52486002
Neurosyphilis	26039008
Non-suppurative otitis media	275481002
Non-tuberculous mycobacterial pneumonia	277869007
Orbital cellulitis	194005002
Orbital osteomyelitis	65875003
Osteomyelitis	60168000

Osteomyelitis of ankle AND/OR foot	28769004
Osteomyelitis of forearm	17028003
Osteomyelitis of hand	61585002
Osteomyelitis of lower leg	21120002
Osteomyelitis of multiple sites	91538002
Osteomyelitis of pelvic region	8872002
Osteomyelitis of shoulder region	53453008
Osteomyelitis of upper arm	75286007
Otitis externa	3135009
Otitis media	65363002
Parainfluenza virus pneumonia	64917006
Pelvic peritonitis	788010000
Peritonitis	48661000
Peritonsillar abscess	15033003
Pneumococcal meningitis	51169003
Pneumococcal peritonitis	58710009
Pneumococcal pneumonia	233607000
Pneumonia	233604007
Pneumonia and influenza	195878008
Pneumonia caused by Chlamydiaceae	233609002
Pneumonia caused by Influenza A virus	772839003
Pneumonia due to anaerobic bacteria	409664000
Pneumonia due to Escherichia coli	51530003
Pneumonia due to Gram negative bacteria	430395005
Pneumonia due to H1N1 influenza	142931000119100
Pneumonia due to infection by Streptococcus pyogenes	441942006
Pneumonia due to methicillin resistant Staphylococcus aureus	124691000119101
Pneumonia due to methicillin susceptible Staphylococcus aureus	128711000119106
Pneumonia due to Pseudomonas	41381004
Pneumonia due to respiratory syncytial virus	195881003
Pneumonia due to Streptococcus	34020007
Pneumonia in aspergillosis	111900000
Primary bacterial peritonitis	11836002
Purulent otitis media	39288006
Purulent peritonitis	235983003
Pyelonephritis	45816000
Pyogenic arthritis of hand	61112000
Pyogenic arthritis of multiple sites	34934005
Pyogenic arthritis of pelvic region	19375000
Pyogenic arthritis of shoulder region	36678001
Pyogenic arthritis of the ankle and/or foot	267882003
Salmonella gastroenteritis	42338000
Salmonella osteomyelitis	6803002
Severe acute respiratory syndrome	398447004
Staphylococcal meningitis	12166008
Staphylococcal pneumonia	22754005
Streptococcal meningitis	4510004

Streptococcal sore throat	43878008
Suppurative arthritis	372939007
Syphilitic endocarditis of aortic valve	278480000
Tonsillitis	90176007
Tuberculosis of hip	14188007
Tuberculosis of kidney	44323002
Tuberculosis of knee	17136009
Tuberculosis of meninges	58437007
Tuberculous peritonitis	44572005
Tuberculous pneumonia	80003002
Upper respiratory tract infection due to H1N1 influenza	142941000119109
Upper respiratory tract infection due to Influenza	10685111000119100
Upper respiratory tract infection due to Influenza A	328531000119104
Urinary tract infectious disease	68566005
Valvular endocarditis	89736004
Ventilator associated pneumonia	429271009
Viral encephalitis	34476008
Viral infection of central nervous system	302810003
Viral meningitis	58170007
Viral pneumonia	75570004
West Nile encephalitis	392662004

SNOMED- Systematized Nomenclature of Medicine

Table S2: List of chronic medical conditions included in analysis by SNOMED codes.

Name	Code
Acute leukemia in remission	91854005
Acute lymphoid leukemia in remission	91856007
Acute lymphoid leukemia relapse	12301000132103
Acute myeloid leukemia in remission	91860005
Acute myeloid leukemia, disease	91861009
Acute myocardial infarction	57054005
Acute myocardial infarction of anterior wall	54329005
Acute myocardial infarction of anterolateral wall	70211005
Acute myocardial infarction of inferior wall	73795002
Acute myocardial infarction of inferolateral wall	65547006
Acute myocardial infarction of inferoposterior wall	76593002
Acute myocardial infarction of lateral wall	58612006
Acute subendocardial infarction	70422006
Asthma	195967001
Asthma-chronic obstructive pulmonary disease overlap syndrome	10692761000119100
Basal cell carcinoma of ear	402820007
Basal cell carcinoma of eyelid	231832009
Basal cell carcinoma of face	402519009
Basal cell carcinoma of lower extremity	402522006

Basal cell carcinoma of skin	254701007
Basal cell carcinoma of skin of lip	402819001
Basal cell carcinoma of truncal skin	402523001
Basal cell carcinoma of upper extremity	402521004
Benign essential hypertension	1201005
Benign hypertensive heart disease with congestive cardiac failure	194767001
Benign secondary hypertension	194785008
Benign secondary renovascular hypertension	73410007
Burkitt's lymphoma (clinical)	118617000
Burkitt's lymphoma of intra-abdominal lymph nodes	188512009
Burkitt's lymphoma of intrapelvic lymph nodes	188515006
Burkitt's lymphoma of lymph nodes of multiple sites	188517003
Burkitt's lymphoma of spleen	188516007
Burkitt's tumor of lymph nodes of head, face AND/OR neck	92512000
Carcinoid syndrome	35868009
Cataract due to diabetes mellitus	43959009
Chronic bronchitis	63480004
Chronic combined systolic and diastolic heart failure	153941000119100
Chronic diastolic heart failure	441530006
Chronic ischemic heart disease	413838009
Chronic kidney disease	709044004
Chronic kidney disease due to hypertension	104931000119100
Chronic kidney disease stage 1	431855005
Chronic kidney disease stage 2	431856006
Chronic kidney disease stage 3	433144002
Chronic kidney disease stage 4	431857002
Chronic kidney disease stage 5	433146000
Chronic kidney disease stage 5 due to hypertension	129161000119100
Chronic leukemia	92812005
Chronic leukemia in remission	92811003
Chronic lymphoid leukemia in remission	92813000
Chronic lymphoid leukemia, disease	92814006
Chronic monocytic leukemia	188745007
Chronic myeloid leukemia	92818009
Chronic myeloid leukemia in remission	92817004
Chronic obstructive lung disease	13645005
Chronic systolic heart failure	441481004
Coma due to diabetes mellitus	420662003
Complication due to diabetes mellitus	74627003
Congestive heart failure	42343007
Congestive rheumatic heart failure	82523003
Cough variant asthma	409663006
Diabetes mellitus	73211009
Diabetes mellitus without complication	111552007
Diastolic heart failure	418304008

Diffuse large B-cell lymphoma (nodal/systemic with skin involvement)	404148006
Disorder due to type 1 diabetes mellitus	420868002
Disorder due to type 2 diabetes mellitus	422014003
Disorder of eye due to diabetes mellitus	25093002
Disorder of eye due to type 1 diabetes mellitus	739681000
Disorder of kidney due to diabetes mellitus	127013003
Disorder of lipid metabolism	267431006
Disorder of nervous system due to diabetes mellitus	422088007
Disorder of nervous system due to type 1 diabetes mellitus	421468001
Emphysematous bronchitis	185086009
End-stage renal disease	46177005
Essential hypertension	59621000
Essential thrombocythemia	109994006
Familial hyperchylomicronemia	267435002
Hairy cell leukemia (clinical)	118613001
Heart failure	84114007
Hodgkin's disease (clinical)	118599009
Hodgkin's disease of intrapelvic lymph nodes	93521006
Hodgkin's disease of intrathoracic lymph nodes	93522004
Hodgkin's disease of lymph nodes of head, face AND/OR neck	93524003
Hodgkin's disease of lymph nodes of inguinal region AND/OR lower limb	93525002
Hodgkin's disease of lymph nodes of multiple sites	93526001
Hodgkin's disease of spleen	93527005
Hodgkin's disease, lymphocytic depletion of lymph nodes of head, face AND/OR neck	93488004
Hodgkin's disease, lymphocytic-histiocytic predominance of intra-abdominal lymph nodes	93493001
Hodgkin's disease, lymphocytic-histiocytic predominance of lymph nodes of axilla AND/OR upper limb	93496009
Hodgkin's disease, lymphocytic-histiocytic predominance of lymph nodes of inguinal region AND/OR lower limb	93498005
Hodgkin's disease, lymphocytic-histiocytic predominance of lymph nodes of multiple sites	188562004
Hodgkin's disease, lymphocytic-histiocytic predominance of spleen	93500006
Hodgkin's disease, mixed cellularity of lymph nodes of head, face AND/OR neck	93506000
Hodgkin's disease, nodular sclerosis (clinical)	118608000
Hodgkin's disease, nodular sclerosis of intrathoracic lymph nodes	188566001
Hodgkin's disease, nodular sclerosis of lymph nodes of head, face AND/OR neck	93515007
Hodgkin's disease, nodular sclerosis of lymph nodes of multiple sites	188572001

Hodgkin's granuloma of lymph nodes of head, face AND/OR neck	93533001
Hyperlipidemia	55822004
Hypertensive disorder	38341003
Hypertensive heart disease with congestive heart failure	5148006
Idiopathic pulmonary fibrosis	700250006
IgE-mediated allergic asthma	424643009
Interstitial emphysema of lung	77690003
Intrinsic asthma	266361008
Kaposi's sarcoma (clinical)	109385007
Kaposi's sarcoma of lung	109390005
Kaposi's sarcoma of soft tissue	188029000
Left heart failure	85232009
Leukemia	93143009
Leukemia in remission	93142004
Leukemic reticuloendotheliosis of extranodal AND/OR solid organ site	93152000
Leukemic reticuloendotheliosis of lymph nodes of head, face and neck	188645002
Lymphoid leukemia	188725004
Lymphoid leukemia in relapse	122951000119108
Lymphoid leukemia in remission	93169003
Lymphosarcoma	188498009
Lymphosarcoma and reticulosarcoma	188487008
Lymphosarcoma of intra-abdominal lymph nodes	188502002
Lymphosarcoma of intrathoracic lymph nodes	188501009
Lymphosarcoma of lymph nodes of head, face and neck	188500005
Lymphosarcoma of lymph nodes of multiple sites	188507008
Malignant carcinoid tumor	445238008
Malignant carcinoid tumor of colon	726654006
Malignant carcinoid tumor of kidney	713574000
Malignant carcinoid tumor of rectum	713573006
Malignant carcinoid tumor of small intestine	709517003
Malignant carcinoid tumor of stomach	709830006
Malignant essential hypertension	78975002
Malignant histiocytosis (clinical)	118612006
Malignant hypertensive end stage renal disease	285841000119104
Malignant hypertensive heart disease with congestive heart failure	83105008
Malignant lymphoma	118600007
Malignant lymphoma of intra-abdominal lymph nodes	93191005
Malignant lymphoma of intrapelvic lymph nodes	93192003
Malignant lymphoma of intrathoracic lymph nodes	93193008
Malignant lymphoma of lymph nodes of axilla AND/OR upper limb	93194002
Malignant lymphoma of lymph nodes of head, face AND/OR neck	93195001

Malignant lymphoma of lymph nodes of inguinal region AND/OR lower limb	93196000
Malignant lymphoma of lymph nodes of multiple sites	93197009
Malignant lymphoma of spleen	93198004
Malignant mast cell tumor (clinical)	118615008
Malignant melanoma of ear and/or external auditory canal	188032002
Malignant melanoma of scalp and/or neck	188044004
Malignant melanoma of skin	93655004
Malignant melanoma of skin of external auditory canal	93222003
Malignant melanoma of skin of eyelid	93224002
Malignant melanoma of skin of face	93225001
Malignant melanoma of skin of lower limb	93641007
Malignant melanoma of skin of trunk	93651008
Malignant melanoma of skin of upper limb	93653006
Malignant neoplasm of abdomen	188366002
Malignant neoplasm of anterior mediastinum	449224009
Malignant neoplasm of anterior wall of urinary bladder	188242006
Malignant neoplasm of auditory tube, middle ear and mastoid air cells	187833006
Malignant neoplasm of axillary tail of female breast	188156001
Malignant neoplasm of brain	428061005
Malignant neoplasm of breast lower inner quadrant	373080008
Malignant neoplasm of breast lower outer quadrant	373081007
Malignant neoplasm of breast upper inner quadrant	373082000
Malignant neoplasm of breast upper outer quadrant	373083005
Malignant neoplasm of central part of female breast	188151006
Malignant neoplasm of cerebral meninges	363474009
Malignant neoplasm of exocervix	372099007
Malignant neoplasm of frontal lobe	363467004
Malignant neoplasm of lateral wall of urinary bladder	188241004
Malignant neoplasm of lip, oral cavity and pharynx	271323007
Malignant neoplasm of liver	93870000
Malignant neoplasm of liver and intrahepatic bile ducts	187767006
Malignant neoplasm of long bone of lower limb	449627008
Malignant neoplasm of lower lobe of lung	187870002
Malignant neoplasm of main bronchus	372065009
Malignant neoplasm of mandible	448668007
Malignant neoplasm of nasal cavities, middle ear and accessory sinuses	187828007
Malignant neoplasm of nervous system	372063002
Malignant neoplasm of occipital lobe	363470000
Malignant neoplasm of parietal lobe	363469001
Malignant neoplasm of pelvic bones, sacrum and coccyx	187952001
Malignant neoplasm of pituitary gland and craniopharyngeal duct	188339002
Malignant neoplasm of posterior wall of urinary bladder	188243001

Malignant neoplasm of rectum, rectosigmoid junction and anus	187760008
Malignant neoplasm of ribs and/or sternum and/or clavicle	187920001
Malignant neoplasm of scalp and/or skin of neck	188102008
Malignant neoplasm of scapula and long bones of upper arm	187929000
Malignant neoplasm of skeletal system	443679004
Malignant neoplasm of skin of eyelid	423425006
Malignant neoplasm of temporal lobe	363468009
Malignant neoplasm of thorax	188361007
Malignant neoplasm of tongue, tip and lateral border	187637005
Malignant neoplasm of upper lobe, bronchus or lung	269464000
Malignant neoplasm of urinary organ	448233000
Malignant neoplasm of vertebral column	363438000
Malignant neoplastic disease	363346000
Malignant neoplastic disease	363346000
Malignant neuroendocrine tumor	133531000119104
Malignant secondary hypertension	89242004
Malignant secondary renovascular hypertension	194783001
Malignant tumor of abdominal part of esophagus	187724003
Malignant tumor of ampulla of Vater	363417006
Malignant tumor of appendix	363411007
Malignant tumor of ascending colon	363412000
Malignant tumor of biliary tract	363415003
Malignant tumor of bladder neck	188244007
Malignant tumor of body of pancreas	187791002
Malignant tumor of body of stomach	187742008
Malignant tumor of breast	254837009
Malignant tumor of colon	363406005
Malignant tumor of conjunctiva	363463000
Malignant tumor of descending colon	363409003
Malignant tumor of esophagus	363402007
Malignant tumor of extrahepatic bile duct	363416002
Malignant tumor of eyelid	231829006
Malignant tumor of fallopian tube	363444001
Malignant tumor of fundus of stomach	187741001
Malignant tumor of glans penis	363451005
Malignant tumor of hard palate	363387004
Malignant tumor of hypopharynx	363399006
Malignant tumor of Islets of Langerhans	187794005
Malignant tumor of jejunum	363404008
Malignant tumor of laryngeal cartilage	363431006
Malignant tumor of larynx	363429002
Malignant tumor of lateral wall of nasopharynx	363398003
Malignant tumor of lower third of esophagus	187727005
Malignant tumor of lymphoid hemopoietic and related tissue	269475001
Malignant tumor of maxillary sinus	363425008
Malignant tumor of middle third of esophagus	187726001

Malignant tumor of nasopharynx	187692001
Malignant tumor of oral cavity	363505006
Malignant tumor of orbit	363462005
Malignant tumor of oropharynx	363392002
Malignant tumor of pancreas	363418001
Malignant tumor of pancreatic duct	187793004
Malignant tumor of parathyroid gland	363481002
Malignant tumor of peritoneum	363492001
Malignant tumor of pituitary gland	363482009
Malignant tumor of pylorus	187736009
Malignant tumor of rectum	363351006
Malignant tumor of renal pelvis	363457009
Malignant tumor of salivary gland	255072001
Malignant tumor of sigmoid colon	363410008
Malignant tumor of small intestine	363509000
Malignant tumor of soft palate	363388009
Malignant tumor of splenic flexure	363413005
Malignant tumor of stomach	363349007
Malignant tumor of submandibular gland	363380002
Malignant tumor of supraglottis	187842004
Malignant tumor of tail of pancreas	187792009
Malignant tumor of testis	363449006
Malignant tumor of thoracic part of esophagus	187723009
Malignant tumor of tongue	363375006
Malignant tumor of tonsillar fossa	363394001
Malignant tumor of tonsillar pillar	187675005
Malignant tumor of transverse colon	363408006
Malignant tumor of ureter	363458004
Malignant tumor of ureteric orifice	188245008
Malignant tumor of urinary bladder	399326009
Malignant tumor of vault of bladder	188240003
Malignant tumor of vermilion border of lower lip	363373004
Malignant tumor of vulva	363367000
Mantle cell lymphoma	443487006
Mantle cell lymphoma of spleen	441559006
Marginal zone lymphoma	447100004
Marginal zone lymphoma of spleen	116691000119101
Merkel cell carcinoma	253001006
Merkel cell carcinoma of lower limb	133881000119100
Merkel cell carcinoma of upper limb	133871000119103
Metastatic carcinoid tumor	705176003
Mixed hyperlipidemia	267434003
Monocytic leukemia	188744006
Multiple myeloma	109989006
Multiple myeloma in remission	94704006
Mycosis fungoides (clinical)	118618005
Mycosis fungoides of extranodal AND/OR solid organ site	94715001

Mycosis fungoides of lymph nodes of multiple sites	188627002
Myelodysplastic syndrome (clinical)	109995007
Myelodysplastic syndrome with isolated del(5q)	277597005
Myeloid leukemia	188732008
Myeloid leukemia in remission	94716000
Myeloid sarcoma	94719007
Myeloid sarcoma in remission	94718004
Myocardial infarction	22298006
Neuroendocrine carcinoma of appendix	717916003
Nodular lymphoma of intra-abdominal lymph nodes	95186006
Nodular lymphoma of intrathoracic lymph nodes	95188007
Nodular lymphoma of lymph nodes of axilla and upper limb	188612002
Nodular lymphoma of lymph nodes of head, face and neck	188609000
Nodular lymphoma of lymph nodes of inguinal region and lower limb	188613007
Nodular lymphoma of lymph nodes of multiple sites	95192000
Nodular lymphoma of spleen	95193005
Nonproliferative retinopathy due to diabetes mellitus	390834004
Obstructive sleep apnea syndrome	78275009
Old myocardial infarction	1755008
Peripheral circulatory disorder due to type 1 diabetes mellitus	421365002
Peripheral T-cell lymphoma (clinical)	109977009
Peripheral vascular disorder due to diabetes mellitus	421895002
Plasma cell leukemia	95210003
Polycythemia vera (clinical)	109992005
Polyneuropathy due to diabetes mellitus	49455004
Primary central nervous system lymphoma	307649006
Primary malignant neoplasm	372087000
Primary malignant neoplasm of abdomen	148911000119107
Primary malignant neoplasm of abdominal esophagus	371962007
Primary malignant neoplasm of adrenal gland	93665005
Primary malignant neoplasm of ampulla of Vater	371967001
Primary malignant neoplasm of anal canal	93669004
Primary malignant neoplasm of anterior aspect of epiglottis	93670003
Primary malignant neoplasm of anterior mediastinum	93671004
Primary malignant neoplasm of anterior two-thirds of tongue	371968006
Primary malignant neoplasm of anterior wall of nasopharynx	93674007
Primary malignant neoplasm of anterior wall of urinary bladder	93675008
Primary malignant neoplasm of anus	93676009
Primary malignant neoplasm of appendix	93679002
Primary malignant neoplasm of ascending colon	93683002
Primary malignant neoplasm of axillary tail of breast	372092003
Primary malignant neoplasm of biliary tract	371970002
Primary malignant neoplasm of bladder	93689003
Primary malignant neoplasm of body of pancreas	93715005
Primary malignant neoplasm of body of stomach	93717002
Primary malignant neoplasm of body of uterus	371971003

Primary malignant neoplasm of bone	93725000
Primary malignant neoplasm of brain	93727008
Primary malignant neoplasm of brain stem	93726004
Primary malignant neoplasm of cecum	371977004
Primary malignant neoplasm of central portion of female breast	93745008
Primary malignant neoplasm of cerebellum	93746009
Primary malignant neoplasm of cerebral meninges	93747000
Primary malignant neoplasm of choroid	93755007
Primary malignant neoplasm of colon	93761005
Primary malignant neoplasm of commissure of lip	371981004
Primary malignant neoplasm of conjunctiva of eye	93764002
Primary malignant neoplasm of descending colon	93771007
Primary malignant neoplasm of dome of urinary bladder	449803009
Primary malignant neoplasm of dorsal surface of tongue	93773005
Primary malignant neoplasm of endocrine gland	371983001
Primary malignant neoplasm of esophagus	371984007
Primary malignant neoplasm of ethmoidal sinus	93787005
Primary malignant neoplasm of exocervix	93789008
Primary malignant neoplasm of extrahepatic bile duct	446189008
Primary malignant neoplasm of eye	371986009
Primary malignant neoplasm of face	93792007
Primary malignant neoplasm of fallopian tube	371987000
Primary malignant neoplasm of female breast	93796005
Primary malignant neoplasm of female genital organ	93797001
Primary malignant neoplasm of floor of mouth	93802007
Primary malignant neoplasm of frontal lobe	93807001
Primary malignant neoplasm of fundus of stomach	93809003
Primary malignant neoplasm of gastrointestinal tract	363745004
Primary malignant neoplasm of glans penis	371989002
Primary malignant neoplasm of greater curvature of stomach	93818001
Primary malignant neoplasm of gum	371990006
Primary malignant neoplasm of hard palate	371991005
Primary malignant neoplasm of head	93824007
Primary malignant neoplasm of heart	93825008
Primary malignant neoplasm of hypopharyngeal aspect of aryepiglottic fold	93829002
Primary malignant neoplasm of hypopharynx	93831006
Primary malignant neoplasm of ileum	93832004
Primary malignant neoplasm of inner aspect of lower lip	93836001
Primary malignant neoplasm of intra-abdominal organs	93839008
Primary malignant neoplasm of intrahepatic bile duct	447109003
Primary malignant neoplasm of intrathoracic organs	93841009
Primary malignant neoplasm of islets of Langerhans	93843007
Primary malignant neoplasm of isthmus of uterus	93844001
Primary malignant neoplasm of jejunum	93846004
Primary malignant neoplasm of labia majora	93850006

Primary malignant neoplasm of lacrimal drainage system	423195009
Primary malignant neoplasm of lacrimal gland	371993008
Primary malignant neoplasm of large intestine	93854002
Primary malignant neoplasm of laryngeal cartilage	109370001
Primary malignant neoplasm of larynx	371995001
Primary malignant neoplasm of lateral portion of floor of mouth	93860002
Primary malignant neoplasm of lateral wall of nasopharynx	93861003
Primary malignant neoplasm of lateral wall of oropharynx	93862005
Primary malignant neoplasm of lateral wall of urinary bladder	93863000
Primary malignant neoplasm of lesser curvature of stomach	93867004
Primary malignant neoplasm of lingual tonsil	93868009
Primary malignant neoplasm of lip	371996000
Primary malignant neoplasm of long bone of lower limb	93871001
Primary malignant neoplasm of lower gum	371997009
Primary malignant neoplasm of lower inner quadrant of female breast	93874009
Primary malignant neoplasm of lower limb	93875005
Primary malignant neoplasm of lower lobe, bronchus or lung	372110008
Primary malignant neoplasm of lower outer quadrant of female breast	93876006
Primary malignant neoplasm of lower third of esophagus	371998004
Primary malignant neoplasm of main bronchus	93882009
Primary malignant neoplasm of major salivary gland	93883004
Primary malignant neoplasm of male breast	93884005
Primary malignant neoplasm of male genital organ	93885006
Primary malignant neoplasm of mandible	93886007
Primary malignant neoplasm of maxillary sinus	93889000
Primary malignant neoplasm of Meckel's diverticulum	93890009
Primary malignant neoplasm of mediastinum	93891008
Primary malignant neoplasm of middle third of esophagus	371999007
Primary malignant neoplasm of nasopharynx	226521000119108
Primary malignant neoplasm of nervous system	93923002
Primary malignant neoplasm of occipital lobe	93928006
Primary malignant neoplasm of oral cavity	372001002
Primary malignant neoplasm of orbit	93932000
Primary malignant neoplasm of oropharynx	93933005
Primary malignant neoplasm of ovary	93934004
Primary malignant neoplasm of palate	372002009
Primary malignant neoplasm of pancreas	372003004
Primary malignant neoplasm of pancreatic duct	93939009
Primary malignant neoplasm of parathyroid gland	93943008
Primary malignant neoplasm of parietal lobe	93946000
Primary malignant neoplasm of pelvic bones, sacrum and coccyx	372115003
Primary malignant neoplasm of pelvis	93953009
Primary malignant neoplasm of penis	372005006
Primary malignant neoplasm of pineal gland	93962006

Primary malignant neoplasm of placenta	721567004
Primary malignant neoplasm of pleura	93966009
Primary malignant neoplasm of posterior mediastinum	93969002
Primary malignant neoplasm of posterior wall of nasopharynx	93970001
Primary malignant neoplasm of posterior wall of urinary bladder	93972009
Primary malignant neoplasm of prostate	93974005
Primary malignant neoplasm of pyloric antrum	93976007
Primary malignant neoplasm of pylorus	93977003
Primary malignant neoplasm of rectum	93984006
Primary malignant neoplasm of renal pelvis	93985007
Primary malignant neoplasm of respiratory tract	93986008
Primary malignant neoplasm of retina	93987004
Primary malignant neoplasm of retromolar area	93989001
Primary malignant neoplasm of ribs and/or sternum and/or clavicle	372107001
Primary malignant neoplasm of scrotum	372009000
Primary malignant neoplasm of short bone of lower limb	94003005
Primary malignant neoplasm of short bone of upper limb	94004004
Primary malignant neoplasm of sigmoid colon	94006002
Primary malignant neoplasm of skin	94047004
Primary malignant neoplasm of skin head and neck	372123001
Primary malignant neoplasm of skin of ear	94017001
Primary malignant neoplasm of skin of eyelid	94021008
Primary malignant neoplasm of skin of face	94022001
Primary malignant neoplasm of skin of lower limb	94033003
Primary malignant neoplasm of skin of trunk	94043000
Primary malignant neoplasm of skin of upper limb	94045007
Primary malignant neoplasm of small intestine	94048009
Primary malignant neoplasm of soft palate	94049001
Primary malignant neoplasm of soft tissues	372010005
Primary malignant neoplasm of soft tissues of abdomen	94050001
Primary malignant neoplasm of soft tissues of lower limb	94057003
Primary malignant neoplasm of soft tissues of pelvis	94059000
Primary malignant neoplasm of soft tissues of thorax	94062002
Primary malignant neoplasm of soft tissues of trunk	94063007
Primary malignant neoplasm of soft tissues of upper limb	372012002
Primary malignant neoplasm of sphenoidal sinus	94067008
Primary malignant neoplasm of spinal cord	94068003
Primary malignant neoplasm of spinal meninges	94069006
Primary malignant neoplasm of spleen	94071006
Primary malignant neoplasm of splenic flexure of colon	94072004
Primary malignant neoplasm of stomach	372014001
Primary malignant neoplasm of submaxillary gland	94077005
Primary malignant neoplasm of supraglottis	94080006
Primary malignant neoplasm of tail of pancreas	94082003
Primary malignant neoplasm of temporal lobe	94086000

Primary malignant neoplasm of testis	94087009
Primary malignant neoplasm of the peritoneum	372016004
Primary malignant neoplasm of thoracic esophagus	372017008
Primary malignant neoplasm of thyroid gland	94098005
Primary malignant neoplasm of tongue	94101009
Primary malignant neoplasm of tonsillar fossa	94102002
Primary malignant neoplasm of tonsillar pillar	94103007
Primary malignant neoplasm of transverse colon	94105000
Primary malignant neoplasm of trunk	94111002
Primary malignant neoplasm of unspecified site (clinical)	109356001
Primary malignant neoplasm of upper inner quadrant of female breast	94115006
Primary malignant neoplasm of upper limb	94116007
Primary malignant neoplasm of upper limb bones and scapula	372133009
Primary malignant neoplasm of upper lobe, bronchus or lung	372135002
Primary malignant neoplasm of upper outer quadrant of female breast	94117003
Primary malignant neoplasm of upper respiratory tract	94118008
Primary malignant neoplasm of upper third of esophagus	372023003
Primary malignant neoplasm of urachus	94120006
Primary malignant neoplasm of ureter	94121005
Primary malignant neoplasm of ureteric orifice of urinary bladder	94122003
Primary malignant neoplasm of urethra	94123008
Primary malignant neoplasm of urinary bladder neck	94124002
Primary malignant neoplasm of urinary system	94125001
Primary malignant neoplasm of uterine adnexa	94126000
Primary malignant neoplasm of uterine cervix	372024009
Primary malignant neoplasm of uterus	10708511000119100
Primary malignant neoplasm of uvula	94129007
Primary malignant neoplasm of ventral surface of tongue	94134006
Primary malignant neoplasm of vermilion border of lip	94135007
Primary malignant neoplasm of vermilion border of lower lip	372026006
Primary malignant neoplasm of vertebral column	372028007
Primary malignant neoplasm of vulva	94143002
Primary malignant neuroendocrine neoplasm of duodenum	721644006
Proliferative retinopathy due to diabetes mellitus	59276001
Pulmonary emphysema	87433001
Pure hypercholesterolemia	267432004
Pure hyperglyceridemia	267433009
Refractory anemia (clinical)	109996008
Refractory anemia with excess blasts (clinical)	398623004
Relapsing chronic myeloid leukemia	415287001
Renal disorder due to type 1 diabetes mellitus	421893009
Renovascular hypertension	123799005
Reticulosarcoma of intra-abdominal lymph nodes	95224004
Reticulosarcoma of intrathoracic lymph nodes	95226002

Secondary diabetes mellitus	8801005
Secondary hypertension	31992008
Secondary malignant neoplasm of adrenal gland	94161006
Secondary malignant neoplasm of bone	94222008
Secondary malignant neoplasm of brain and spinal cord	188462001
Secondary malignant neoplasm of female breast	94297009
Secondary malignant neoplasm of intra-abdominal organs	94348003
Secondary malignant neoplasm of large intestine	94365007
Secondary malignant neoplasm of liver	94381002
Secondary malignant neoplasm of lymph node	94392001
Secondary malignant neoplasm of mediastinum	94409002
Secondary malignant neoplasm of nervous system	94442001
Secondary malignant neoplasm of ovary	94455000
Secondary malignant neoplasm of pleura	94493005
Secondary malignant neoplasm of respiratory and digestive systems	269473008
Secondary malignant neoplasm of respiratory tract	94515004
Secondary malignant neoplasm of retroperitoneum and peritoneum	188445006
Secondary malignant neoplasm of skin	94579000
Secondary malignant neoplasm of small intestine	94580002
Secondary malignant neoplasm of trunk	94649002
Secondary malignant neoplasm of urinary system	94663008
Secondary malignant neoplastic disease	128462008
Severe asthma	370221004
Sezary's disease	118611004
Squamous cell carcinoma of eyelid	231831002
Squamous cell carcinoma of skin	254651007
Squamous cell carcinoma of skin of ear	403894000
Squamous cell carcinoma of skin of face	403892001
Squamous cell carcinoma of skin of lower extremity	403898002
Squamous cell carcinoma of skin of lower lip	285308002
Squamous cell carcinoma of skin of trunk	403899005
Squamous cell carcinoma of upper extremity	403897007
Subacute lymphoid leukemia	188726003
Subacute myeloid leukemia	188736006
Systolic heart failure	417996009
True posterior myocardial infarction	194802003
Type 1 diabetes mellitus	46635009
Type 1 diabetes mellitus without complication	313435000
Type 2 diabetes mellitus	44054006
Type 2 diabetes mellitus without complication	313436004
Widespread metastatic malignant neoplastic disease	405843009

SNOMED- Systematized Nomenclature of Medicine

Table S3: Descriptive statistics and outcomes. Analysis without underweight patients.

BMI categories	Normal 18.5-24.9	Overweight 25-29.9	Obesity I 30-34.9	Obesity II 35-39.9	Obesity III 40-45	p
n	9307	8702	4179	1612	611	
age (mean (SD))	58.24 (24.04)	63.41 (19.58)	63.68 (18.20)	63.15 (17.69)	61.64 (16.57)	<0.001
Male (%)	4674 (50.2)	4829 (55.5)	2019 (48.3)	630 (39.1)	234 (38.3)	<0.001
BMI (mean (SD))	22.42 (1.72)	27.29 (1.38)	32.09 (1.38)	37.01 (1.38)	42.05 (1.44)	<0.001
**Comorbidities**						
MetS (%)	685 (7.4)	910 (10.5)	492 (11.8)	201 (12.5)	68 (11.1)	<0.001
HTN (%)	512 (5.5)	712 (8.2)	419 (10.0)	159 (9.9)	64 (10.5)	<0.001
Hyperlipidemia (%)	359 (3.9)	545 (6.3)	295 (7.1)	122 (7.6)	45 (7.4)	<0.001
DM (%)	1724 (18.5)	2400 (27.6)	1397 (33.4)	666 (41.3)	276 (45.2)	<0.001
CLD (%)	1164 (12.5)	1210 (13.9)	695 (16.6)	363 (22.5)	155 (25.4)	<0.001
IHD (%)	1363 (14.6)	1669 (19.2)	816 (19.5)	318 (19.7)	130 (21.3)	<0.001
HF (%)	926 (9.9)	1146 (13.2)	591 (14.1)	315 (19.5)	142 (23.2)	<0.001
Malignancy (%)	1450 (15.6)	1379 (15.8)	621 (14.9)	245 (15.2)	69 (11.3)	0.034
CKD (%)	762 (8.2)	830 (9.5)	407 (9.7)	193 (12.0)	72 (11.8)	<0.001
**Infectious disease**						
Endocarditis (%)	47 (0.5)	51 (0.6)	18 (0.4)	7 (0.4)	3 (0.5)	0.797
Peritonitis (%)	139 (1.5)	126 (1.4)	41 (1.0)	19 (1.2)	9 (1.5)	0.158
Diverticulitis (%)	202 (2.2)	324 (3.7)	156 (3.7)	42 (2.6)	17 (2.8)	<0.001
Appendicitis (%)	1150 (12.4)	727 (8.4)	280 (6.7)	94 (5.8)	16 (2.6)	<0.001
Biliary infection* (%)	400 (4.3)	528 (6.1)	235 (5.6)	93 (5.8)	30 (4.9)	<0.001
Pneumonia (%)	2416 (26.0)	2297 (26.4)	1051 (25.1)	370 (23.0)	132 (21.6)	0.005
Viral URTI (%)	475 (5.1)	578 (6.6)	309 (7.4)	108 (6.7)	48 (7.9)	<0.001
ENT infection (%)	602 (6.5)	391 (4.5)	194 (4.6)	70 (4.3)	32 (5.2)	<0.001
CNS infection (%)	169 (1.8)	103 (1.2)	35 (0.8)	14 (0.9)	2 (0.3)	<0.001
Necrotizing fasciitis (%)	6 (0.1)	10 (0.1)	6 (0.1)	5 (0.3)	1 (0.2)	0.097
Infective arthritis (%)	4 (0.0)	12 (0.1)	5 (0.1)	0 (0.0)	1 (0.2)	0.151
Osteomyelitis (%)	55 (0.6)	57 (0.7)	10 (0.2)	11 (0.7)	2 (0.3)	0.034
Cellulitis (%)	529 (5.7)	639 (7.3)	387 (9.3)	212 (13.2)	87 (14.2)	<0.001
UTI (%)	1637 (17.6)	1451 (16.7)	718 (17.2)	241 (15.0)	72 (11.8)	0.001

**Laboratory results**						
Albumin (mean (SD))	34.11 (5.64)	34.62 (5.20)	35.10 (4.82)	34.54 (4.82)	34.87 (4.62)	<0.001
Creatinine (mean (SD))	1.11 (1.00)	1.16 (0.95)	1.19 (0.93)	1.24 (1.09)	1.21 (1.06)	<0.001
CRP (mean (SD))	95.16 (92.50)	97.95 (93.65)	100.30 (96.56)	104.27 (103.09)	106.62 (102.78)	<0.001
Hemoglobin (mean (SD))	12.41 (2.07)	12.58 (2.07)	12.62 (2.00)	12.34 (2.05)	12.37 (1.99)	<0.001
**Outcomes**						
90-day mortality (%)	1227 (13.2)	774 (8.9)	300 (7.2)	145 (9.0)	43 (7.0)	<0.001
1-year mortality (%)	1916 (20.6)	1356 (15.6)	544 (13.0)	242 (15.0)	92 (15.1)	<0.001
5-year mortality (%)	3052 (32.8)	2556 (29.4)	1103 (26.4)	487 (30.2)	167 (27.3)	<0.001

Table S4: Survival probability by BMI group up to 5-years after hospitalization with an infectious disease. Analysis without underweight patients.

	**HR**	**95% CI**		***p***
age	1.05	1.05	1.05	<0.001
Male	1.13	1.08	1.19	<0.001
Overweight (25-29.9)	0.76	0.72	0.80	<0.001
Obesity I (30-34.9)	0.71	0.66	0.76	<0.001
Obesity II (35-39.9)	0.77	0.70	0.85	<0.001
Obesity III (40-45)	0.78	0.67	0.92	0.003
Albumin	0.91	0.91	0.92	<0.001
Creatinine	1.11	1.09	1.13	<0.001
CRP	1.00	1.00	1.00	<0.001
Hemoglobin	0.92	0.91	0.94	<0.001
DM	1.15	1.10	1.21	<0.001
CLD	1.22	1.15	1.29	<0.001
IHD	1.03	0.97	1.09	0.358
Past MI	1.05	0.98	1.14	0.181
HF	1.42	1.34	1.50	<0.001
Malignancy	1.39	1.32	1.46	<0.001
CKD	1.01	0.94	1.08	0.782
Endocarditis	1.23	0.95	1.60	0.122
Peritonitis	0.91	0.76	1.10	0.334
Diverticulitis	0.40	0.31	0.52	<0.001
Appendicitis	0.27	0.20	0.36	<0.001
Biliary infection*	0.74	0.66	0.84	<0.001

Pneumonia	1.26	1.19	1.33	<0.001
Viral URTI	0.97	0.87	1.09	0.594
ENT infection	0.88	0.70	1.10	0.263
CNS infection	0.98	0.69	1.37	0.884
Necrotizing fasciitis	1.04	0.64	1.68	0.887
Infective arthritis	0.38	0.17	0.86	0.019
Osteomyelitis	1.18	0.92	1.50	0.184
Cellulitis	0.97	0.88	1.07	0.490
UTI	0.97	0.90	1.04	0.355
MetS	1.18	1.01	1.37	0.036
HTN	0.99	0.85	1.15	0.910
Hyperlipidemia	0.88	0.78	0.99	0.040
